# Genome-Wide Profiling Reveals the Landscape of Prognostic Alternative Splicing Signatures in Pancreatic Ductal Adenocarcinoma

**DOI:** 10.3389/fonc.2019.00511

**Published:** 2019-06-18

**Authors:** Chengkun Yang, Qiongyuan Wu, Ketuan Huang, Xiangkun Wang, Tingdong Yu, Xiwen Liao, Jianlu Huang, Guangzhi Zhu, Yizhen Gong, Chuangye Han, Hao Su, Wei Qin, Tao Peng

**Affiliations:** ^1^Department of Hepatobiliary Surgery, the First Affiliated Hospital of Guangxi Medical University, Nanning, China; ^2^Department of Tuina, the First Affiliated Hospital of Guangxi University of Chinese Medicine, Nanning, China; ^3^Department of Colorectal and Anal Surgery, the First Affiliated Hospital of Guangxi Medical University, Nanning, China

**Keywords:** PDAC, alternative splicing, prognosis, splicing factor, correlation

## Abstract

Pancreatic ductal adenocarcinoma (PDAC) is an aggressive lethal malignancy. Identification of potential alternative splicing (AS) prognostic indicators and related splicing pathways for the prediction of PDAC outcomes is lacking but urgently needed. A combined strategy of prognostic assessment and computational biology was performed to investigate survival-related AS signatures and their correlation with splicing factors. The prognostic signatures of each type were conducted according to the top 10 prognosis-related AS events, which were filtered through univariate Cox regression analysis. A time-dependent receiver operating characteristic curve was constructed to access the predictive accuracy of prognostic signatures. The independent predictors were identified using multivariate Cox regression analysis. Potential regulation mechanisms between splicing factors and splicing events were investigated through regulatory networks and correlation analyses. A total of 915 overall survival (OS) and 480 recurrence-free survival (RFS)-related AS events were identified in 120 patients with PDAC. The independent prognostic signatures for each type displayed favorable accuracy for the prediction of OS and short-term RFS [area under the curves were >0.6] except for the Exclusive Exons type. The splicing regulatory networks showed potential interactions between splicing factors and AS parent genes. Moreover, a positive relationship was detected among each splicing factor and Percent Spliced In values of prognostic signatures. Our results provide a view of the landscape of prognosis-related AS events and reveal the potential correlation between splicing factors and prognostic signatures, which may represent novel outcome-predictor markers and opportunities for targeted therapy for PDAC.

## Introduction

Pancreatic cancer is a lethal malignancy with a 5 years survival rate of <6% due to its aggressive biological characteristics and the lack of effective treatment (1). The latest reports show that pancreatic cancer is a leading cause of cancer-related death among middle-aged and elderly people, both males and females, in the United States (2). It has been reported that 91,000 cases of pancreatic cancer were diagnosed in 2015 and that 79,400 individuals died as a result of this disease in China, where pancreatic cancer is ranked 9 and 6th in cancer incidence and mortality, respectively (3). In addition, only about 15% of patients are candidates for curative surgery, and patients who undergo attempts at curative surgery plus adjuvant chemotherapy still have a very poor 5 years survival rate of ~15–20%, with 80% of patients facing recurrence after pancreatectomy (4–6). Pancreatic ductal adenocarcinoma (PDAC) is the most common histological type of pancreatic cancer. The poor prognosis of pancreatic cancer is closely related to its local recurrence, lymph nodes and early metastasis (7, 8). These poor outcomes highlight an urgent need to develop novel predictive biomarkers for patient prognosis and treatment response, with potential linkage to distinct therapeutic options.

Alternative splicing (AS) is one of the key regulatory mechanisms leading to transcriptome and proteome diversity that result from the generation of alternative mRNA transcripts and the encoding of a series of structurally and functionally distinct protein isoforms that can have different types of biological activity (9). It has been estimated that approximately 95% of multi-exon human genes undergo AS events (10). The application of genome-scale RNA sequencing greatly promotes the identification of AS in tumors. AS is currently categorized into 7 transition types: Alternate Acceptor site (AA); Alternate Donor site (AD); Alternate Promoter (AP); Alternate Terminator (AT); Exon Skip (ES); Retained Intron (RI); and Mutually Exclusive Exons (ME). A growing number of studies have demonstrated that aberrant AS events provide potential molecular markers for malignancies (11–13). Dysregulation of AS creates phenotypic complexity and also leads to aberrant protein isoforms, which may contribute to many oncogenic processes and difficulties for therapeutic treatments (10, 12, 13). Available evidence reveals that AS events are associated with more than 20% of disease-causing single base-pair mutations (14). Genome-wide transcriptome analysis is sensitive in detecting key biological mechanisms of carcinogenesis, de-differentiation and metastasis in multiple tissue types. Improved understanding of shifts in splicing patterns often provide better treatment responses and patients' prognosis prediction.

The rapid development of high-throughput technologies has disclosed that altered AS events are involved in the mutation of RNA sequences and the aberrant expression of RNA-binding proteins. Dysregulation of splicing factors has been frequently observed and accounts for numerous altered AS events that occur in different cancer types (15–18). Splicing factors play a crucial role in the regulation of splicing for spliceosome assembly. Different expression levels of splicing factors have been observed to affect the splicing patterns of many genes that function in certain cancer-associated biological pathways (19, 20). Considering the close association between AS events and splicing factor gene expression, and the fact that they are only superficially recognized, it is essential to explore the regulatory mechanism and look into the possibility of developing new therapies for PDAC.

Recently, the prognostic value of AS signatures has been identified in multiple cancer types, including ovarian cancer (21), non-small cell lung cancer (22), and colorectal cancer (23). However, few comprehensive studies have detailed PDAC-specific AS events with their prognostic value in a genome-wide transcriptome approach. Therefore, we conducted a systematic profile of prognosis-related AS in PDAC using genome-wide AS events and corresponding clinical information. Our results provide the landscape of AS signatures with various predictive accuracies. In addition, further investigation of splicing factors with their potential precise targets may shed new light on fully understanding the contribution of genetic variants in tumorigenesis and development.

## Materials and Methods

### Overview of This Study Design and Processing Details

**Figure 1:**
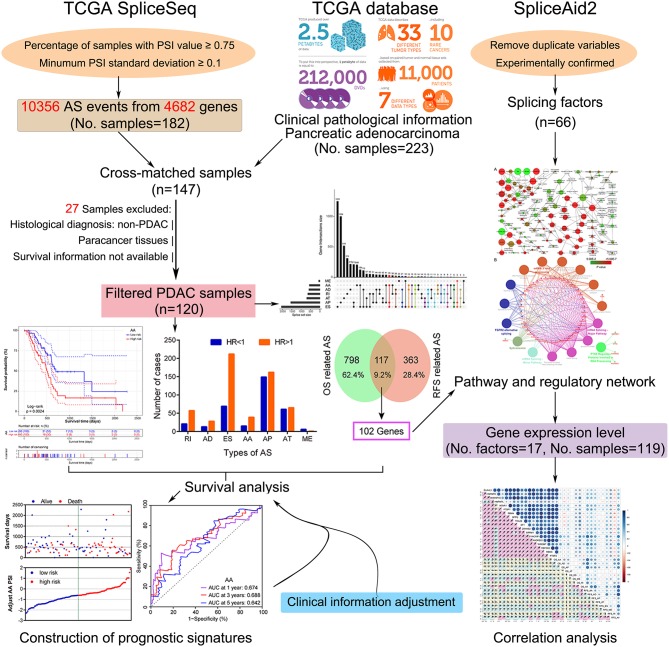
Flowchart of the systematic profiling of alternative splicing in pancreatic ductal adenocarcinoma in the present study. TCGA, The Cancer Genome Atlas; AS, alternative splicing; PDAC, pancreatic ductal adenocarcinoma; OS, overall survival; RFS, recurrence-free survival.

In this study, we employed a combination strategy of prognostic assessment and computational biology to investigate the survival-related AS signatures and their correlation with splicing factors. A flow chart summarizing the present work is shown in Figure 1. Firstly, after selection using a series of stringent filters, prognostic signatures of each AS type were implemented in patients with PDAC. Subsequently, enrichment and correlation analysis were applied to identify the potential association between splicing factors and prognostic signatures.

### Data Acquisition and Curation Pre-processing

RNA sequencing expression data and corresponding clinical information of the pancreatic adenocarcinoma dataset in The Cancer Genome Atlas (TCGA) was downloaded from the University of California, Santa Cruz Xena browser (UCSC Xena: http://xena.ucsc.edu/, accessed November 23, 2018). Percent Spliced In (PSI) values of AS events in pancreatic adenocarcinoma samples were obtained from TCGA SpliceSeq (24) (http://bioinformatics.mdanderson.org/TCGASpliceSeq, accessed November 25, 2018), a web-based resource for exploring the AS patterns of TCGA tumors. The inclusion criteria were required samples with a PSI value of ≥0.75 and a standard deviation of >0.10. A total number of 147 PDAC patients were matched after referencing their sample ID and clinical information. Then, 27 samples were excluded due to the following filters: (i) Pathological type was non-PDAC (*n* = 10); (ii) Paracancer tissues (non-tumor tissues; *n* = 16); and (iii) Survival information not available (*n* = 1). Finally, 120 samples with 10,356 AS events and 4,682 parent genes were enrolled in the survival analysis.

In order to explore the potential affection of splicing factors on prognostic AS signatures, a list of splicing factors was extracted from the SpliceAid2 database (25) (www.introni.it/spliceaid.html, accessed November 28, 2018), a curated online repository of human RNA target motifs bound by splicing proteins. After the removal of duplicates, 66 experimentally validated splicing factors were further analyzed.

### Survival Analysis and Prognostic Signature Construction

Univariate Cox regression analysis was used to calculate the relationships between the PSI values of AS events and the prognosis of patients. The median AS PSI value was set as the cutoff value for categorizing all patients into a high or low percentage group for survival analysis. Processing was done using the *Survival* R package (26) (https://github.com/therneau/survival). A Venn diagram was used to present relationships between interactive sets of overall survival (OS) and recurrence-free survival (RFS)-related AS events. Circos plots were generated using the *Circlize* package (27) to display details of the AS events and the genes involved in a genome-wide distribution of human genomic features.

A prognosis risk score was determined based on the linear combination of AS PSI multiplied by a corresponding regression coefficient (β) representingthe weight of the correlation. The regression coefficient was calculated from a univariate Cox proportional hazards regression model. Then, each prognostic AS event was fitted to the univariate Cox regression model, with clinical outcomes as a dependent variable. The risk score formula was as follows: risk score = PSI of AS_1_ × β_1_AS_1_ + PSI of AS_2_ × β_2_AS_2_ +…PSI of AS_n_ × β_n_AS_n_ (28, 29). The top 10 most significant AS events among the seven types (except ME which was <10) were chosen as survival-related factors and were used to establish candidate prognostic signatures. Patients were divided into high and low risk groups according to their median risk score.

Kaplan–Meier survival analysis was conducted to analyze the difference between the two groups. Survival curves were compared with the log-rank test. A time-dependent receiver operating characteristic (ROC) curve, constructed using the *survivalROC* package (30), was carried out to assess the predictive accuracy of prognostic signatures in patients with PDAC. The area under the curve (AUC) of the ROC curve was calculated for each prognostic signature. Multivariate Cox analysis was employed to determine the possibility of signatures being independent risk predictors after clinical characteristics were adjusted.

### Construction of the Splicing Correlation Network

Gene ontology analysis for biological processes, cellular components, and molecular functions were investigated using the Biological Networks Gene Ontology tool (BiNGO) (31). The Kyoto encyclopedia of genes and genomes, and the Reactome pathway enrichment analyses were facilitated by ClueGO (32), setting the pathways with a *p* < 0.01 and pathway network connectivity (Kappa score) of >0.7. All regulatory networks were constructed using Cytoscape (version 3.5.1).

Correlation analyses were employed to determine whether the expression of these splicing factor genes was significantly associated with the PSI values of survival related AS signatures. Pearson's correlation coefficient was calculated to evaluate co-expression relationships among the genes and assess the potential associations between the expression level of splicing factors and the PSI value of the AS events.

### Statistical Analyses

All statistical analyses were performed using SPSS version 24.0 (IBMCorp., Chicago, IL, USA) and R 3.4.1 (http://www.r-project.org/). The intersections and aggregates between different types of AS were visualized using the *UpSetR* package (33). Hazard ratios (HRs) and 95% confidence intervals (CIs) were used to assess relative risk of PDAC patients with different PSI values of AS events and of different risk groups. Gene to gene interactions were investigated using GeneMANIA (34) (http://www.genemania.org/, accessed December 5, 2018). The survival status distributions of different risks were plotted by GraphPad Prism 5.01 (GraphPad Software, Inc., San Diego, CA, USA). All statistical tests with a two-sided *p* < 0.05 were considered significant.

## Results

### Overview of as Event Profiling and Distribution in PDAC

**Figure 2:**
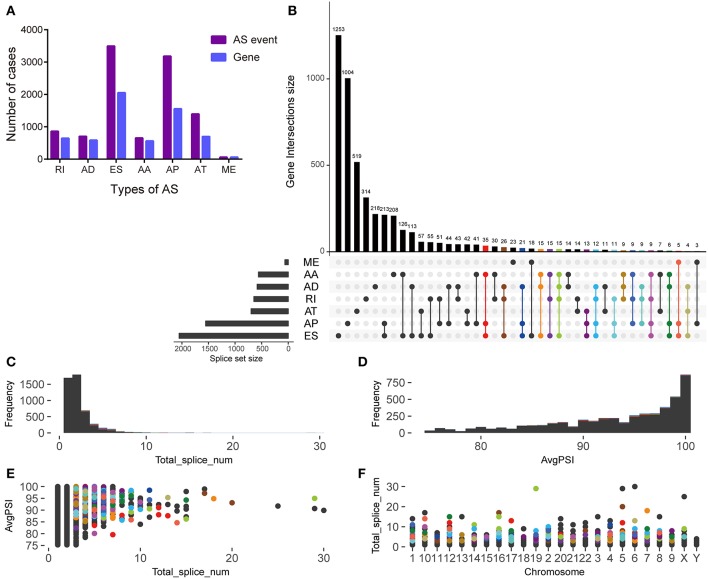
Overview of alternative splicing (AS) event profiling and genes involved in pancreatic ductal adenocarcinoma. **(A)** The number of different AS types and genes involved in patients with pancreatic ductal adenocarcinoma. **(B)** UpSet plot of interactions between alternative splicing events and its parent genes. **(C)** The frequency distribution of genes carrying more than three different AS types for total events. **(D)** The frequency distribution of genes carrying more than three different AS types for an average PSI. **(E)** The scatter plot of genes carrying more than three different AS types and an average AS PSI. **(F)** The distribution of genes carrying more than three different AS types in chromosomes.

The histogram shows the total number of AS events and corresponding genes in different AS types (Figure 2A). Among them, ES and AP events were the highest in number. The UpSet plot displays the interaction between genes and different AS types (Figure 2B). One gene may have up to four types of AS and >45% of genes (*n* = 688) contain two or more AS events. However, in this study a low proportion of genes (*n* = 210) was found to carry more than three splice sites (Figure 2C). After screening, most of the enrolled AS events had a PSI of more than 90% (Figure 2D). The scatter plots show the distribution of the genes carrying more than three splice sites at different PSI values and chromosomes (Figures 2E,F).

### Identification of Survival-Related as Events

**Figure 3:**
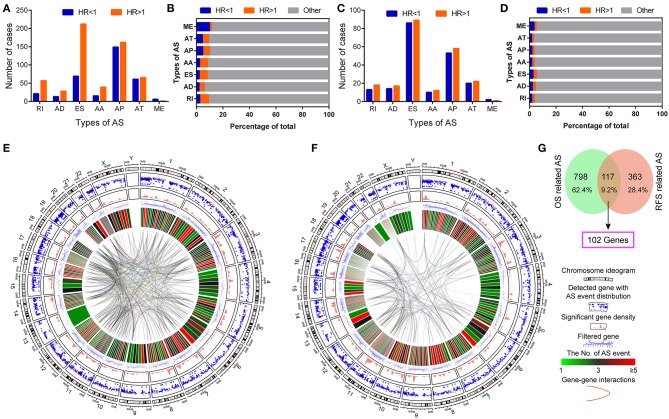
Identification of survival related AS events in pancreatic ductal adenocarcinoma. **(A,B)** The number and proportion of favorable OS-related (HR < 1) and adverse OS-related (HR > 1) AS events in pancreatic ductal adenocarcinoma. **(C,D)** The number and proportion of favorable RFS-related (HR < 1) and adverse RFS-related (HR > 1) AS events in pancreatic ductal adenocarcinoma. **(E,F)** Circos Plots of detailed AS events and its parent genes in chromosomes. Circos panels from outside to the inside are represented as follows: the genomic axes, Rainfall plot, genomic density, filtered parent genes, number of AS events, and gene to gene interactions. **(G)** Venn plot of prognosis-related alternative splicing events and the overlap of genes.

Univariate survival analysis identified favorable and adverse prognosis-related AS events. The total number and proportion of OS and RFS-related AS events were 915 (HR < 1: 582, 5.62%; HR > 1: 333, 3.22%) and 480 (HR < 1: 248, 2.39%; HR > 1: 232, 2.24%), respectively (Figures 3A–D, Table S1). The Circos plots demonstrate the details of prognosis-related AS events of the genes involved (Figures 3E,F). The intersection between OS and RFS-related AS events show 117 events and 102 parent genes were detected in PDAC (Figure 3G).

### Construction of Prognostic Signatures

**Figure 4:**
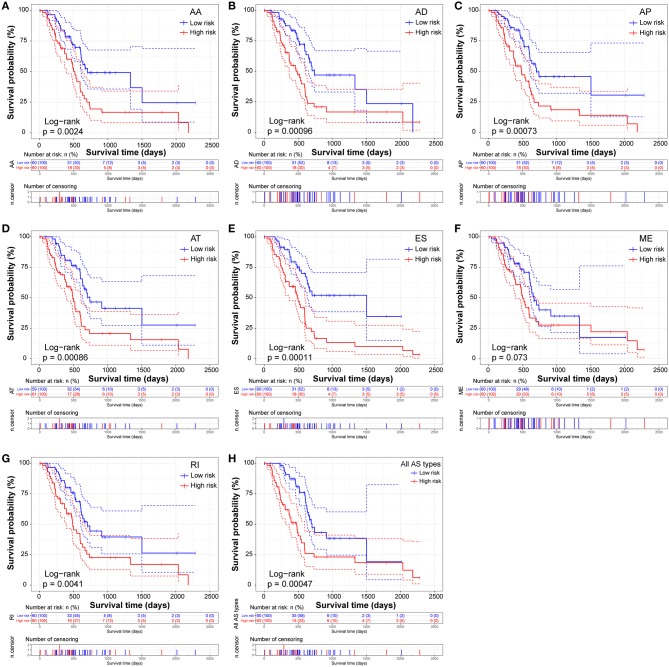
Kaplan-Meier Survival analysis of overall survival (OS)-related predictors in seven types of AS events. Kaplan-Meier curves for **(A)** Alternate Acceptor site (AA); **(B)** Alternate Donor site (AD); **(C)** Alternate Promoter (AP); **(D)** Alternate Terminator (AT); **(E)** Exon Skip (ES); **(F)** Mutually Exclusive Exons (ME); **(G)** Retained Intron (RI); and **(H)** All types of AS. Survival curves assessed using the log-rank test.

**Figure 5:**
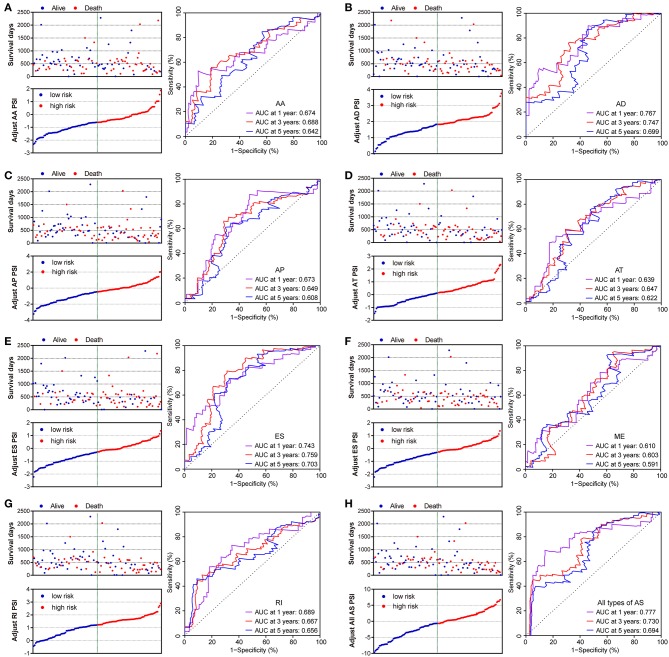
Distribution of risk stratification and time-dependent receiver operating characteristic (ROC) curves of OS-related predictors in patients with pancreatic ductal adenocarcinoma. Dot plots for patients with different risk and ROC curves for different survival times. **(A)** Alternate Acceptor site (AA); **(B)** Alternate Donor site (AD); **(C)** Alternate Promoter (AP); **(D)** Alternate Terminator (AT); **(E)** Exon Skip (ES); **(F)** Mutually Exclusive Exons (ME); **(G)** Retained Intron (RI); and **(H)** All types of AS.

The prognostic signature of each AS type was established based on risk scores. The risk score formulas are summarized in Table S2. Kaplan–Meier survival analysis was conducted to assess the relationship between the signatures and prognosis. The results suggest that seven AS prognostic signatures (AA, AD, AP, AT, ES, RI, and all AS) were significantly associated with the OS of patients with PDAC (all *p* < 0.005, Figures 4A–E,G,H). However, there was no statistical difference between two groups of the ME prognostic signature (*p* = 0.073, Figure 4F). The survival status distribution of the high and low risk groups of prognostic signatures are displayed in Figure 5. Most AUCs of the time-dependent ROC for the eight prognostic signatures were more than 0.6 in value (Figure 5). In order to identify the independent prognostic indicator, multivariate Cox regression analysis was performed on different prognostic signatures adjusted with age, gender, tumor stage, residual tumor status, targeted molecular therapy, and radiation therapy. The association of clinical pathological features and clinical outcomes is shown in Table S3. After adjustment, eight prognostic signatures were able to serve as independent risk predictors for the OS of patients with PDAC (all adjusted *p* < 0.05, Table 1).

**Table 1:** Survival analysis of prognostic predictors in pancreatic ductal adenocarcinoma.

**AS events**	**E/N** **(Low)**	**MST** **(Low)**	**E/N** **(High)**	**MST** **(High)**	**UniCox HR** **(95% CI, H vs. L)**	**Log-Rank *P***	**MultiCox HR (95% CI, H vs. L)**	**Adjusted *P***
**OS**								
AA	25/60	702	39/60	518	2.15 (1.30–3.56)	0.002	2.03 (1.15–3.59)	0.015
AD	25/60	738	39/60	486	2.30 (1.38–3.81)	0.001	3.46 (1.81–6.62)	< 0.001
AP	24/60	702	40/60	481	2.35 (1.41–3.91)	0.001	2.30 (1.23–4.31)	0.010
AT	24/60	738	40/60	486	2.53 (1.51–4.23)	< 0.001	2.68 (1.48–4.85)	0.001
ES	22/60	1502	42/60	486	2.71 (1.61–4.58)	< 0.001	3.39 (1.86–6.16)	< 0.001
ME	27/60	666	37/60	498	1.58 (0.95–2.63)	0.073	1.86 (1.06–3.26)	0.031
RI	26/60	702	38/60	486	2.06 (1.24–3.40)	0.004	2.04 (1.12–3.72)	0.019
All AS	25/60	702	39/60	476	2.44 (1.46–4.08)	< 0.001	2.80 (1.38–5.69)	0.004
**RFS**								
AA	15/60	1210	35/60	390	3.76 (2.03–6.95)	< 0.001	5.22 (2.42–11.26)	< 0.001
AD	12/60	1600	38/60	439	4.28 (2.18–8.40)	< 0.001	3.41 (1.69–6.89)	0.001
AP	18/59	831	32/61	513	1.99 (1.11–3.55)	0.024	2.29 (1.21–4.36)	0.011
AT	18/59	1309	32/61	486	2.58 (1.43–4.66)	0.002	2.74 (1.38–5.42)	0.004
ES	16/60	872	34/60	439	2.60 (1.43–4.73)	0.001	2.36 (1.22–4.59)	0.011
ME	23/59	593	27/61	542	1.34 (0.77–2.35)	0.419	1.87 (0.97–3.58)	0.060
RI	18/60	872	32/60	443	2.30 (1.28–4.11)	0.004	3.00 (1.55–5.81)	0.001
All AS	13/60	1210	37/60	375	4.93 (2.60–9.36)	< 0.001	11.20 (4.38–28.62)	< 0.001

*Multivariate Cox proportional hazards regression model was adjusted by age, gender, tumor stage, residual tumor status, targeted molecular therapy, and radiation therapy*.

**Figure 6:**
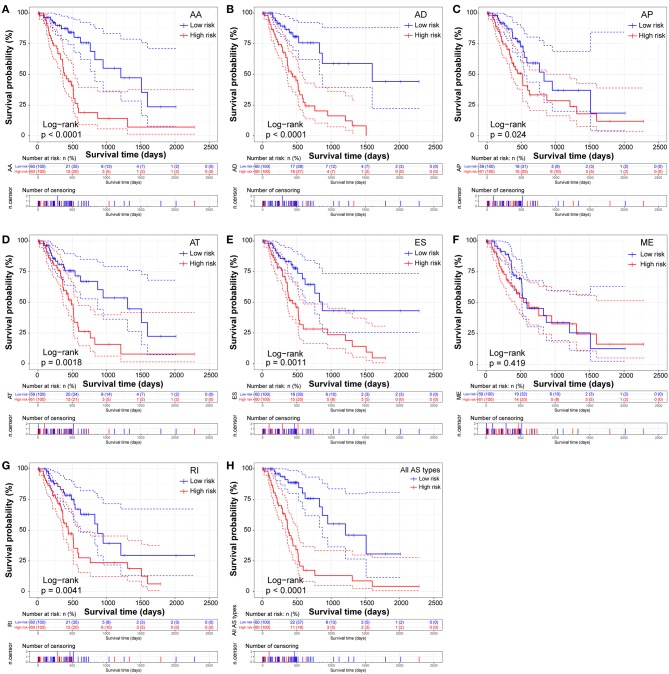
Kaplan-Meier Survival analysis of recurrence-free survival (RFS)-related predictors in seven types of AS events. Kaplan-Meier curves for **(A)** Alternate Acceptor site (AA); **(B)** Alternate Donor site (AD); **(C)** Alternate Promoter (AP); **(D)** Alternate Terminator (AT); **(E)** Exon Skip (ES); **(F)** Mutually Exclusive Exons (ME); **(G)** Retained Intron (RI); and **(H)** All types of AS. Survival curves assessed using the log-rank test.

**Figure 7:**
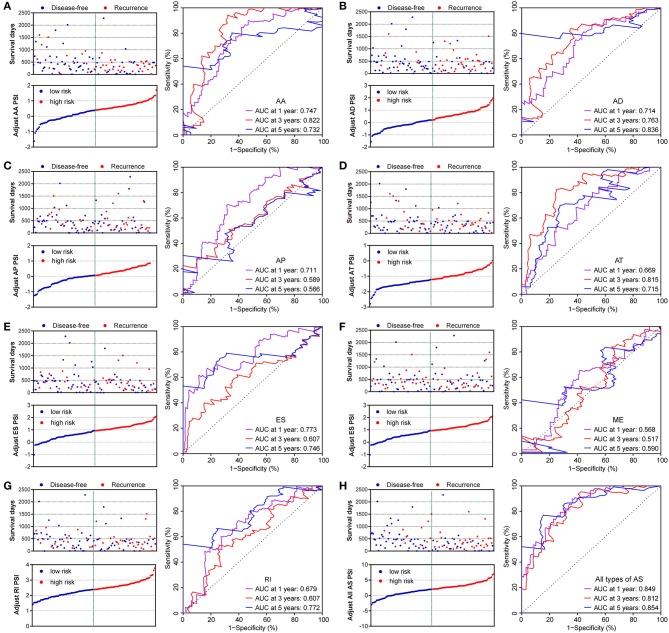
Distribution of risk stratification and time-dependent receiver operating characteristic (ROC) curve of RFS-related predictors in patients with pancreatic ductal adenocarcinoma. Dot plots for patients with different risk and ROC curves for different survival times. **(A)** Alternate Acceptor site (AA); **(B)** Alternate Donor site (AD); **(C)** Alternate Promoter (AP); **(D)** Alternate Terminator (AT); **(E)** Exon Skip (ES); **(F)** Mutually Exclusive Exons (ME); **(G)** Retained Intron (RI); and **(H)** All types of AS.

Similarly, two groups of seven AS prognostic signatures (AA, AD, AP, AT, ES, RI, and all AS) had statistical differences with regard to the RFS of patients with PDAC (all *p* < 0.05, Figures 6A–E,G–H), except for the ME prognostic signature (*p* = 0.419, Figure 6F). Multivariate Cox regression analysis also suggested seven AS prognostic signatures (AA, AD, AP, AT, ES, RI, and all AS) act as independent prognostic indicators for RFS of PDAC patients (all adjusted *P* < 0.05, Table 1). The survival status distribution of high and low risk groups for prognostic signatures are displayed in Figure 7. Most AUCs of the ROC curves for eight prognostic signatures contain a value higher than 0.7 for the short term RFS, implying a favorable predictive accuracy for patients with PDAC after pancreatectomy (Figure 7).

### Pathway Network Construction and Functional Enrichment Analysis Between Splicing Factors and as Parent Genes

**Figure 8:**
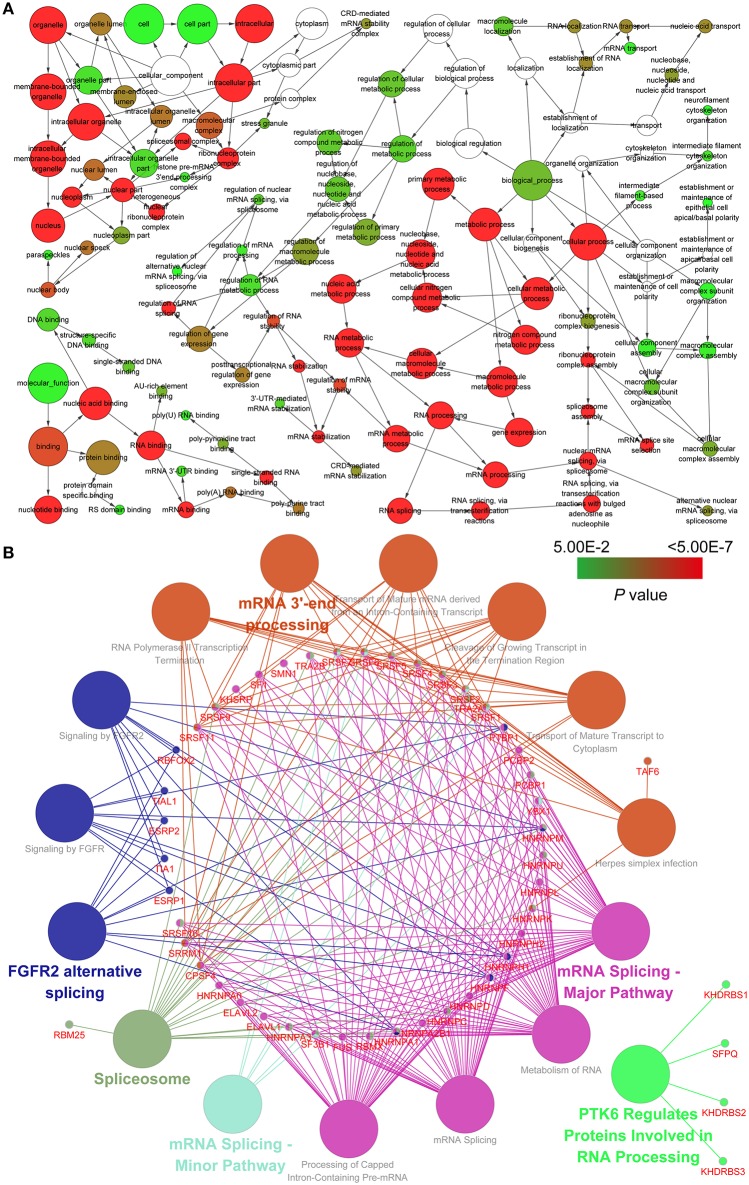
Pathway analysis and the regulation network between splicing factors and survival-related AS events of genes involved. **(A)** Gene ontology analysis for biological processes, cellular components, and molecular functions. **(B)** Kyoto Encyclopedia of Genes and Genomes and Reactome pathway analysis between splicing factors and survival-related AS events of genes involved.

To investigate the potential relationship between splicing factors and AS parent genes, we carried out an enrichment analysis using BiNGO and ClueGO tools. Hierarchical connections and interactions between splicing factors and AS parent genes indicate their roles in multiple biological regulatory activities (Figure 8A; Table S4). Utilizing the pathway enrichment analysis, we detected that splicing factors and AS parent genes play a critical role in mRNA splicing, mRNA ^3′^-end processing and gene regulation (Figure 8B; Table S4).

### Correlation Analysis Between Splicing Factor Gene Expression Levels and the Psi of Prognostic Signatures

**Figure 9:**
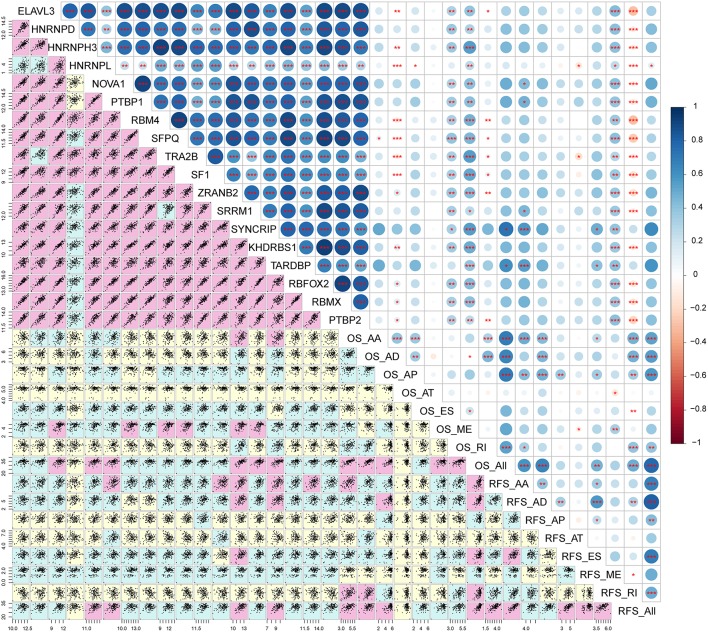
Correlation analysis between splicing factors and AS prognostic predictors. The lower panel of the figure displays scatter plots of the correlation between expression of splicing factors and the PSI values of survival related AS events. Background color depth represents the weight of the correlation coefficient. Pink, green, and yellow show strong, medium and weak correlations, respectively. The upper panel of the figure demonstrates the correlation coefficient and the significance of the correlation between the expression of splicing factors and the PSI values of survival related AS events. The size and color of the circle represent the weight of the correlation coefficient, *p < 0.05; **p < 0.01; ***p < 0.001.

Correlation analysis was performed to explore the candidate regulation network. The result implies that most splicing factor genes are significantly associated with each other (*r* > 0.8, *p* < 0.01, Figure 9; Table S5). In addition, the expression levels of splicing factor genes are weakly positively associated with the PSI of prognostic signatures (Figure 9; Table S5).

## Discussions

In this study, we detected prognosis-related signatures and investigated the association between splicing factors and the PSI of signatures. In humans, different combinations of AS events provide perhaps the largest potential process for expanding transcriptome and proteome diversity. The functions altered by AS are varied and include biomass generation, induction of angiogenesis, loss of genomic stability and deterioration of the immune system (35). Recent evidence indicates that some of these can be used as prognostic or diagnostic biomarkers, and the development of strategies to correct and/or inhibit pathological splicing events will be key steps in developing future therapeutic approaches (36). Thus, the application of prognosis-related AS events as potential targets for cancer therapy need to be urgently investigated.

Our results of the overview of AS events in patients with PDAC demonstrate that nearly half of all genes have two or more AS events emerged, indicating that numerous splicing events may produce disease-specific protein isoforms and the mis-assembly splicing-altering genetic variants. In some diseases, aberrant AS events play a vital role in somatic spliceosome mutations, tumorigenesis and tumor metastasis (37–39). Compared with their normal tissue counterparts, distinct patterns of cancer-specific AS events have been reported for multiple tumors (40). It has been recently reported that altered AS events of angiogenesis and the NOTCH pathway play an important role in the pathogenesis of PDAC, which can serve as an indicators for diagnosis (41). Wang et al. reported that on average there are two AS events in 1,354 significantly identified protein-coding genes, with ES and AP types being the most frequently utilized, and are a group of alternatively spliced genes that encode surface and circulating proteins as novel candidates of potential diagnostic and therapeutic targets of PDAC (42). Due to the limited sample size and lack of clinical data, the researchers did not further explore the relationship between differential AS and prognosis in patients with PDAC. However, our results identified prognosis-related AS signatures as a powerful complement that can be used to verify the link between AS events and PDAC.

Studies have demonstrated that high-risk AS types are associated with poor OS in multiple cancers, and predictive models of each AS type performed reasonably well in distinguishing between good and poor outcomes of patients (21–23). Similarly, we filtered the OS-related AS events in patients with PDAC, using univariate Cox regression analyses, as well as RFS of patients. Our study identified 915 OS-related and 480 RFS-related AS events and assessed their predictive value in HCC prognosis using the top 10 AS events in each type. Adjusted with prognosis-related clinical characteristics, the results of multivariate Cox regression analyses suggest that the OS and RFS-related signatures perform well as independent indicators of PDAC. The time dependent ROC curves present predictive models that harbor a noteworthy ability in distinguishing between the outcome for patients, including OS and short-term RFS, which provides outcome estimation and the opportunity to develop new therapies for PDAC.

In general, splicing factors are a crucial spliceosome catalyst in regulating splicing reactions, a key mechanism of post-transcriptional gene expression regulation. Defects in AS are most frequently identified in human tumors and result either from mutations in splicing regulatory factors of cancer-specific genes or from confusions in the AS regulatory machinery (43). Cancer-specific AS events are intimately linked with tumorigenesis through the regulation of genes affecting tumor proliferation, metastasis and drug resistance (44). In this regard, changes in the expression of some splicing factors have been directly linked to the expression of oncogenic splice variants that confer various advantages to cancer cells (45–47). Our results of clinicopathological characteristics identified that the use of target molecule therapy rapidly decreases the risk of negative clinical outcomes in patients with PDAC. Gemcitabine, currently the most commonly used drug for chemotherapy in PDAC, achieves only minor benefits, as a result of the chemoresistance that stems from the development of chemotherapy escape pathways (48). Gemcitabine induces the expression of the oncogenic splicing factor, which promotes the occurrence of AS events in upstream regulatory pathways, provoking a long-lasting feedback response to therapeutic treatments, and confers increased drug resistance in PDAC cell lines (49). Calabretta et al. reported that chronic gemcitabine treatment led to the isolation of drug-resistant PDAC cells that display higher resistance to gemcitabine and cisplatin, due to confusions in splicing factor expression and the dysregulation of AS events, facilitating the restoration of sensitivity of PDAC cells to drug treatment (50). Therefore, identification of molecular pathways in splicing factors and AS events are proposed as new potential therapeutic targets that may improve the response of PDAC to chemotherapy.

In order to investigate the association between splicing factors and prognosis-related AS events, we carried out gene ontology and pathway enrichment analysis using the tools BiNGO and ClueGO. The angiogenesis and proliferation factor-related pathways and splicing factors were identified in the enrichment analysis, indicating that splicing factors influence oncogenic processes by regulating the AS of many downstream target genes during the relapse of tumors. In addition, a positive relationship was found in the correlation analysis. Studies have reported that splicing factors were associated with proliferation and invasion of multiple tumors (51–54). The findings of this study shed light on better understanding of the role of splicing factors in the splicing machinery of PDAC and can be used to guide the targeting of cancer-specific splicing isoforms as a cancer therapy.

However, there are several limitations in this study that should be considered. Firstly, the total number of PDAC patients included in our study was limited. Secondly, the lack of independent cohorts of patients being used to verify the prognostic models being proposed makes this study not reproducible. Further studies are needed to clarify the role of splicing factors in PDAC, while further functional experiments and clinical trials are needed to identify pathways between splicing factors and AS events.

In summary, our results highlight the prognostic value of AS events and explore potential regulatory mechanisms between splicing factors and prognostic signatures at the genome level. The comprehensive portrait of transcription systematically characterizes the prognostic value of AS, suggesting that the PSI of AS events and its splicing signature are related. Splicing factors represent novel outcome-predictor markers and the development of opportunities for targeted therapies for PDAC.

## Data Availability

All data obtained for this study can be found in TCGA, SpliceSeq and SpliceAid2 databases.

## Ethics Statement

This investigation was approved by the Ethics Committee of the First Affiliated Hospital of Guangxi Medical University.

## Author Contributions

CY and TP designed the study. CY, QW, KH, and XW performed research. XW, XL, JH, and TY provided sample collection and clinical support. GZ, YG, HS, WQ, and CH contributed to data interpretation. CY and QW wrote the manuscript. TP critically revised the manuscript and participated in the analysis and interpretation of the data. All authors reviewed, edited and approved the final version of the manuscript.

### Conflict of Interest Statement

The authors declare that the research was conducted in the absence of any commercial or financial relationships that could be construed as a potential conflict of interest.

## Abbreviations

AA: Alternate Acceptor site
AD: Alternate Donor site
AP: Alternate Promoter
AS: Alternative Splicing
AT: Alternate Terminator
CI: Confidence interval
E: Events
ES: Exon Skip
HR: Hazard ratio
ME: Mutually Exclusive Exons
MST: Median survival time
N: Number
OS: Overall Survival
RFS: Recurrence-free Survival
RI: Retained Intron.
