# Supplementary material for: Genome-Wide Profiling Reveals the Landscape of Prognostic Alternative Splicing Signatures in Pancreatic Ductal Adenocarcinoma
Source: Front Oncol. 2019 Jun 18;9:511. doi: 10.3389/fonc.2019.00511 (PMC6591313; doi:10.3389/fonc.2019.00511)
Supplement: Supplementary file 2 [file Table_2.docx]

Table S2. Construction of prognostic predictors for pancreatic ductal adenocarcinoma.

| **Survival analysis** | **AS events** | **Formula** |
| --- | --- | --- |
| **OS** | AA | PRPF40B_21599_AA*0.9203+SLC25A29_29255_AA*1.0986+ZNF720_36288_AA*(0.9416)+CHORDC1_18268_AA*0.8459  +NCOA1_52827_AA*(-0.9163)+PRELID2_73896_AA*(-0.8916)+ENTPD6_58865_AA*0.8587+TM2D2_83474_AA*(-0.8210)  +TRPV2_39436_AA*(-0.7765)+NSMF_88315_AA*0.8198 |
|  | AD | KIF2A_72179_AD*1.0473+MRPL11_17032_AD*0.8629+RSAD1_42400_AD*0.8629+GTF2H2C_72400_AD*(-0.9676)  +SEMA6C_7562_AD*0.9042+ZNF266_47339_AD*(-0.9416)+HECTD1_27107_AD*0.8242+C4orf48_68544_AD*(-0.9163)  +MRPL43_12856_AD*0.7747+ABHD3_44778_AD*0.7324 |
|  | AP | SBNO2_46390_AP*0.9933+NFKBIZ_65981_AP*1.0613+NFKBIZ_65982_AP*(-1.0498)+NRF1_81751_AP*(-1.0788)  +ME3_18193_AP*(-0.9416)+SDR39U1_27005_AP*0.9322+NPIPB4_34531_AP*0.94+SBNO2_46391_AP*(-0.9163)  +ZNF841_51403_AP*(-0.9163)+ZNF841_51405_AP*0.9042 |
|  | AT | PLEKHM3_57205_AT*(-0.9676)+FANCM_27405_AT*(-0.8916)+FANCM_27406_AT*0.9163+GCOM1_30799_AT*(-0.8916)  +EMR2_48028_AT*0.9042+ZNF549_52284_AT*(-0.8916)+PLEKHM3_57206_AT*0.9439+FMO5_7368_AT*0.8329  +EMR2_48029_AT*(-0.8440)+GCOM1_30798_AT*0.8286 |
|  | ES | MIS18BP1_27410_ES*1.0613+SKA2_42753_ES*(-1.0788)+PIGT_59575_ES*1.3191+GTF2H2_72439_ES*1.3056  +MRPL55_10120_ES*(-0.9163)+ZCCHC10_73332_ES*(-0.9416)+MUC1_121767_ES*(-0.9416)+COL3A1_306292_ES*1.0188  +ODF2L_3675_ES*(-0.8210)+IMMP1L_14817_ES*(-0.8440) |
|  | ME | N4BP2L1_25590_ME*(-0.8210)+C14orf2_29528_ME*(-0.7550)+AKAP13_32352_ME*(-0.6539)  +TMEM104_217418_ME*0.6206+TBC1D5_63665_ME*(-0.6733)+SMPD4_55291_ME*(-0.5798)+RBKS_53050_ME*(-0.6349) |
|  | RI | MAP7D1_1760_RI*(-0.8916)+GTPBP3_48289_RI*0.9163+RBM5_64960_RI*0.9439+TBCB_49357_RI*0.8329  +FAM57A_38258_RI*0.8879+ERRFI1_533_RI*0.7608+SMUG1_22125_RI*(-0.7765)+YPEL3_36075_RI*(-0.7550)  +SPRTN_10277_RI*1.2149+MDM1_22927_RI*1.0367 |
|  | All_AS | AA+AD+AP+AT+ES+ME+RI |
|  |  |  |
| **RFS** | AA | PRPF40B_21599_AA*1.1053+RALGAPB_59379_AA*(-1.0498)+ITIH4_65276_AA*1.0225+ENTPD6_58865_AA*0.8459  +RALGAPB_59380_AA*0.892+MTFP1_61752_AA*(-0.8440)+RPLP0_24727_AA*(-0.7765)+HSF4_36944_AA*0.7701  +MUC1_8020_AA*(-0.7765)+BOLA1_7409_AA*(-1.3471) |
|  | AD | WHSC1L1_83394_AD*1.0043+UBE2I_33057_AD*1.1346+ARL16_44157_AD*(-1.0217)+ELMOD3_54211_AD*(-0.9416)  +TGIF1_44508_AD*(0.9163)+FES_32502_AD*0.9083+STRADA_42981_AD*0.8713+ASB16_41780_AD*0.8838  +NFATC1_46241_AD*(-0.8675)+SULF2_59730_AD*0.8242 |
|  | AP | RAB3D_47651_AP*(-1.1394)+FYN_77272_AP*1.0919+POM121C_80118_AP*0.9933+THAP4_58391_AP*0.9322  +POM121C_80119_AP*(-0.8916)+MEIS2_29906_AP*0.9361+COPS7A_19933_AP*0.9123+RAB3D_47652_AP*0.8796  +RNF44_74655_AP*0.8755+COPS7A_19932_AP*(-0.8916) |
|  | AT | KCTD13_35989_AT*0.9203+ANKRD29_44842_AT*(-0.8440)+KATNAL2_45430_AT*(-0.8210)+FAT3_18279_AT*(-0.8210)  +ERAP1_72865_AT*(-0.8210)+VWA5B1_923_AT*(-0.7985+ASPHD1_35984_AT*(-0.8440)+ACOXL_54943_AT*(-0.8210)  +LLGL2_43460_AT*0.7885+FAM71D_28039_AT*0.8796 |
|  | ES | FAM21A_11563_ES*1.0682+TTLL5_28523_ES*(0.9676)+FAM114A1_69035_ES*(0.9416)+DMKN_49172_ES*1.0367  +TPCN2_101162_ES*1.0116+SENP1_21410_ES*0.94+PPP1R12A_23532_ES*(-0.8210)+THOC5_61613_ES*0.8502  +MEIS1_53815_ES*0.8502+TMX2_15919_ES*0.8065 |
|  | ME | N4BP2L1_25590_ME*(-0.7765)+NDUFAF6_84594_ME*(-0.7765)+STEAP3_95656_ME*0.6419 |
|  | RI | CYB561D2_65050_RI*0.8329+RPS15_46491_RI*0.793+TMPRSS3_60711_RI*0.8154+GNB1L_61083_RI*0.802  +SLC35C1_15509_RI*0.7701+NDUFS7_46465_RI*0.7839+DMAP1_2545_RI*0.7419+CCL4L1_40420_RI*0.8109  +SF3B1_56678_RI*(-0.7340)+ZNF226_50290_RI*0.7178 |
|  | All_AS | AA+AD+AP+AT+ES+ME+RI |

**Notes**: Prognostic predictors were formed by the top 10 of seven types of AS events, except for ME.

**Abbreviation**: AS, Alternative Splicing; OS, Overall Survival; RFS, Recurrence-free Survival; AA, Alternate Acceptor site; AD, Alternate Donor site; AP, Alternate Promoter; AT, Alternate Terminator; ES, Exon Skip; ME, Mutually Exclusive Exons; RI, Retained Intron.
