# Supplementary material for: Genome-Wide Profiling Reveals the Landscape of Prognostic Alternative Splicing Signatures in Pancreatic Ductal Adenocarcinoma
Source: Front Oncol. 2019 Jun 18;9:511. doi: 10.3389/fonc.2019.00511 (PMC6591313; doi:10.3389/fonc.2019.00511)
Supplement: Supplementary file 3 [file Table_3.docx]

Table S3. Clinical features of the patients with pancreatic ductal adenocarcinoma.

| Variables | | N | MST (days) | HR (95%CI) | Log-rank *P* | MRT (days) | HR (95%CI) | Log-rank *P* |
| --- | --- | --- | --- | --- | --- | --- | --- | --- |
| Age (years) | ≤60 | 35 | 607 | 1 |  | 486 | 1 |  |
|  | >60 | 85 | 598 | 1.34 (0.76-2.34) | 0.308 | 593 | 0.71 (0.40-1.25) | 0.231 |
| Gender | female | 56 | 593 | 1 |  | 831 | 1 |  |
|  | male | 64 | 634 | 0.76 (0.46-1.24) | 0.270 | 519 | 1.32 (0.75-2.34) | 0.332 |
| Race | white | 103 | 603 | 1 |  | 593 | 1 |  |
|  | others | 13 | 1332 | 0.60 (0.26-1.40) | 0.231 | 526 | 0.92 (0.41-2.08) | 0.839 |
| History of chronic pancreatitis | No | 84 | 627 | 1 |  | 526 | 1 |  |
|  | Yes | 8 | 607 | 1.20 (0.47-3.04) | 0.701 | 763 | 1.00 (0.31-3.24) | 0.994 |
| History of diabetes | No | 70 | 634 | 1 |  | 521 | 1 |  |
|  | Yes | 27 | 603 | 1.12 (0.57-2.22) | 0.741 | 593 | 0.91 (0.43-1.92) | 0.805 |
| Alcohol exposures | No | 45 | 532 | 1 |  | 831 | 1 |  |
|  | Yes | 64 | 634 | 0.77 (0.46-1.30) | 0.327 | 526 | 1.07 (0.58-1.97) | 0.834 |
| Tumor Stage | I/II | 114 | 603 | 1 |  | 581 | 1 |  |
|  | III/IV | 5 | 545 | 0.73 (0.18-3.00) | 0.663 | NA | 0.65 (0.09-4.70) | 0.664 |
| Pathologic M | M0 | 66 | 598 | 1 |  | 542 | 1 |  |
|  | Mx | 54 | 614 | 0.68 (0.41-1.12) | 0.128 | 763 | 0.74 (0.42-1.32) | 0.307 |
| Pathologic N | N0 | 33 | 1332 | 1 |  | 831 | 1 |  |
|  | N1 | 87 | 593 | 1.68 (0.91-3.09) | 0.093 | 542 | 1.41 (0.75-2.67) | 0.283 |
| Pathologic T | T1 | 16 | 532 | 1 |  | 763 | 1 |  |
|  | T3 | 103 | 607 | 0.88 (0.42-1.85) | 0.735 | 581 | 0.90 (0.40-2.01) | 0.790 |
| Histologic grade | G1/G2 | 88 | 627 | 1 |  | 763 | 1 |  |
|  | G3/G4 | 32 | 517 | 1.40 (0.82-2.38) | 0.210 | 519 | 1.28 (0.69-2.39) | 0.435 |
| Residual tumor | R0 | 68 | 614 | 1 |  | 763 | 1 |  |
|  | R1/Rx | 46 | 532 | 1.61 (0.96-2.71) | 0.068 | 416 | **2.08 (1.14-3.77)** | **0.014** |
| Targeted molecular therapy | No | 27 | 239 | 1 |  | 330 | 1 |  |
|  | Yes | 84 | 691 | **0.17 (0.10-0.31)** | **<0.001** | 620 | **0.40 (0.19-0.83)** | **0.011** |
| Radiation therapy | No | 77 | 532 | 1 |  | 581 | 1 |  |
|  | Yes | 29 | 702 | 0.59 (0.32-1.08) | 0.083 | 620 | 0.78 (0.42-1.47) | 0.446 |
